# Signal sequence-triage is activated by translocon obstruction sensed by an ER stress sensor IRE1α

**DOI:** 10.1247/csf.23072

**Published:** 2023-09-28

**Authors:** Ashuei Sogawa, Ryota Komori, Kota Yanagitani, Miku Ohfurudono, Akio Tsuru, Koji Kadoi, Yukio Kimata, Hiderou Yoshida, Kenji Kohno

**Affiliations:** 1 Laboratory of Molecular and Cell Genetics, Graduate School of Biological Sciences, Nara Institute of Science and Technology (NAIST), 8916-5 Takayama, Ikoma, Nara 630-0192, Japan; 2 Osaka International Cancer Institute (OICI), 3-1-69 Otemae, Chuo-ku, Osaka, Osaka 541-8567, Japan; 3 Institute for Research Initiatives, Nara Institute of Science and Technology (NAIST), 8916-5 Takayama, Ikoma, Nara 630-0192, Japan; 4 Division of Biological Science, Graduate School of Science and Technology, Nara Institute of Science and Technology (NAIST), 8916-5 Takayama, Ikoma, Nara 630-0192, Japan; 5 Department of Biochemistry and Molecular Biology, Graduate School of Science, University of Hyogo, Harima Science Garden City, Hyogo 678-1297, Japan; 6 Ubiquitin Biology Laboratory, Graduate School of Frontier Biosciences, Osaka University, 1-3 Yamadaoka, Suita, Osaka 565-0871, Japan

**Keywords:** endoplasmic reticulum, translocation capacity, translocon clogging, IRE1, signal sequence

## Abstract

Secretory pathway proteins are cotranslationally translocated into the endoplasmic reticulum (ER) of metazoan cells through the protein channel, translocon. Given that there are far fewer translocons than ribosomes in a cell, it is essential that secretory protein-translating ribosomes only occupy translocons transiently. Therefore, if translocons are obstructed by ribosomes stalled or slowed in translational elongation, it possibly results in deleterious consequences to cellular function. Hence, we investigated how translocon clogging by stalled ribosomes affects mammalian cells. First, we constructed ER-destined translational arrest proteins (ER-TAP) as an artificial protein that clogged the translocon in the ER membrane. Here, we show that the translocon clogging by ER-TAP expression activates triage of signal sequences (SS) in which secretory pathway proteins harboring highly efficient SS are preferentially translocated into the ER lumen. Interestingly, the translocon obstructed status specifically activates inositol requiring enzyme 1α (IRE1α) but not protein kinase R-like ER kinase (PERK). Given that the IRE1α–XBP1 pathway mainly induces the translocon components, our discovery implies that lowered availability of translocon activates IRE1α, which induces translocon itself. This results in rebalance between protein influx into the ER and the cellular translocation capacity.

## Introduction

In eukaryotic cells, translational elongation is carried out by 80S ribosomes, which decode the codons in an mRNA molecule and sequentially ligate the corresponding amino acids. This process is complete when the ribosome reaches a termination codon. At the termination codon, the 80S ribosome is split into 60S and 40S ribosomal subunits by eukaryotic release factors (eRF1 and eRF3), enabling the cells to recycle ribosomes. Although translational elongation has been considered a smooth process, the translation on ribosomes can be stalled by various mRNA roadblocks. These comprise RNA-binding proteins, higher-order structural regions of mRNAs, and obstructers of the protein exit tunnel of the translating ribosome. The latter include basic amino acid clusters or specific motifs that interfere with the peptidyl transfer activity of the ribosome (Doma and Parker, 2006; Ito-Harashima *et al.*, 2007; Lu and Deutsch, 2008; Kuroha *et al.*, 2010; Ingolia *et al.*, 2011; Yanagitani *et al.*, 2011; Shanmuganathan *et al.*, 2019).

Ribosomes can be roughly separated into two categories: endoplasmic reticulum (ER) membrane-bound ribosomes that synthesize proteins destined for the secretory pathway (secretory proteins, SPs) and free ribosomes that synthesize all other proteins. Most SPs translocate into the ER lumen via the Sec61 translocon in a cotranslational manner. The estimated copy number of translocons within a single cell is far lower than that of ribosomes (Kulak *et al.*, 2014). Therefore, an SP-translating ribosome must only occupy a translocon for a limited time. If translational arrest occurs during the synthesis of SPs, aberrant ribosomes obstruct the translocon. This results in a reduction in the translocation capacity of the cell.

The quality control mechanism of the nascent polypeptides on the translocon of the ER membrane during a translational arrest has often been analyzed in detail (Izawa *et al.*, 2012; von der Malsburg *et al.*, 2015; Arakawa *et al.*, 2016). On the other hand, it is poorly understood how a cell responds to the translocon obstruction. Here, we report on ER translocation efficiency of SPs and the response of ER stress sensors during the translocon obstructed status in mammalian cells.

## Materials and Methods

### Plasmids

To determine the ability to induce translational arrest, we constructed a series of arrest reporter plasmids, pcDNA3.1(+) GFP-X-RFP. In the constructs, tester sequences were inserted at position “X,” which was located between the enhanced green fluorescent protein (EGFP) and mCherry sequences (Fig. S1A). The exact sequences of the testers and linkers surrounding the testers are shown in Fig. S1B. Note that the C-terminus of mCherry was tagged with HA. The pcDNA3.1(+)_Fluc-ER plasmid has been described previously (Nakamura *et al.*, 2011). To generate Fluc-ER[R30], 30 consecutive arginine (R30) residue-encoding sequences were inserted between the firefly luciferase- and KDEL (Lys-Asp-Glu-Leu; ER retention sequence)-encoding sequences in pcDNA3.1(+)_Fluc-ER using polymerase chain reaction (PCR) and DNA ligation. In the case of Fluc[R30], the SS was deleted from pcDNA3.1_Fluc-ER[R30] by PCR. The exact sequences of R30 and the surrounding sequences are shown in Fig. S2A. pcDNA3.1(+)_α-1-antitrypsin (A1AT) was generated by inserting human A1AT complementary DNA (cDNA) into pcDNA3.1(+). Then, A1AT[PrP] and A1AT[Lep] were generated by substituting the SS of A1AT (amino acids 1 to 24: MPSSVSWGILLLAGLCCLVPVSLA) with that of human prion (amino acids 1 to 22: MANLGCWMLVLFVATWSDLGLC) or human leptin (amino acids 1 to 21: MHWGTLCGFLWLWPYLFYVQA) by PCR and DNA ligation. To generate A1AT[R30], 30 consecutive arginine (R30) residue-encoding sequences were inserted just upstream of the termination codon of A1AT in pcDNA3.1(+)_A1AT using PCR and DNA ligation.

### Antibodies

Anti-firefly luciferase (Abcam, Cambridge, UK; ab187340, 1:4000), anti-GAPDH (CST, Danvers, MA, USA; #2118, 1:1000), anti-calnexin (Stressgen, San Diego, CA, USA; SPA-860, 1:1000), anti-Sec61α (Sigma Aldrich, St. Louis, MO, USA; SAB2500917, 1:1000), anti-β-actin (CST, #4970, 1:1000), anti-FLAG M2 (Sigma Aldrich; F1804, 1:1000), anti-α-1-antitrypsin (DAKO, Glostrup, Denmark; A0012, 1:1000), anti-IRE1α (CST, #3294, 1:1000), anti-PERK (CST, #3192, 1:500), anti-CHOP (CST, #2895T, 1:500), anti-IgG (H+L chain) (Rabbit) pAb-horseradish peroxidase (HRP) (MBL, Tokyo, Japan; 458, 1:5000), peroxidase AffiniPure goat anti-mouse IgG (H+L) (Jackson Immuno Research Laboratories, West Grove, PA, USA; 115-035-003, 1:5000), anti-rabbit IgG, HRP-linked antibody (CST, 7074, 1:3000), and anti-mouse IgG, HRP-linked antibody (CST, 7076, 1:3000) were purchased. Anti-Sec61β (1:1000) was a kind gift from Dr Tom A. Rapoport (Harvard Medical School, Boston, MA, USA). Anti-ERP57 antibody (1:1000) was generated in our laboratory (Sopha *et al.*, 2012).

### *In vitro* transcription and translation

For *in vitro* transcription, pcDNA3.1(+)_GFP-X-RFP was amplified by PCR using the forward primer 5'-ATTTAGGTGACACTATAGAAGAGacccaagctggctagcg-3' (the uppercase letters indicate the SP6 promoter, and the lowercase letters indicate the sequence that hybridizes to pcDNA3.1(+)), and the reverse primer 5'-TTTTTTTTTTTTTTTTTTTTTTTTTTTTTTcacctactcagacaatgcgatgc-3' (the uppercase letters indicate the sequence for the poly A template, and the lowercase letters indicate the sequence that hybridizes to pcDNA3.1(+)). Thermal cycling conditions were as follows: denaturation at 98°C for 5 min, and 30 cycles with denaturation at 98°C for 10 s, and then annealing/elongation at 68°C for 3 min using SimpliAmp^TM^ Thermal Cycler (Applied Biosystems/Life Technologies Japan, ThermoFisher Scientific K.K., Tokyo, Japan). The 5'-capped mRNAs were transcribed from the purified PCR products at 37°C for 2 h according to the manufacturer’s instructions for Riboprobe *in vitro* transcription systems (Promega, Madison, WI, USA). Note that we used SP6 polymerase (Promega) and Ribo m^7^G Cap analogue (Promega) for the transcription. The template DNA was removed using DNase I (TaKaRa, Kusatsu, Japan) at 37°C for 20 min. The synthesized mRNAs were purified using ISOGEN-LS (Nippon Gene, Tokyo, Japan).

The synthesized RNAs were translated at 30°C with an *in vitro* translation system using rabbit reticulocyte lysate (Promega). The synthesized proteins were labelled with ^35^S-labeled methionine and cysteine using the EasyTag^TM^ EXPRE^35^S^35^S protein labelling mix (PerkinElmer, Waltham, MA, USA). To prevent proteasomal degradation of the synthesized proteins, the translational mixtures included 10 μM MG-132. Note that we added 8 μg/mL harringtonin (Santa Cruz Biotechnology, Dallas, TX, USA; sc-204771A) to the mixtures after 5 min of translation. The translation was stopped by freezing with liquid N_2_. The samples were then mixed with lysis buffer A comprising 0.35 M Bis-tris (pH 6.5), 2% sodium dodecyl sulfate (SDS), 10% glycerol, 20 μM MG-132, 1 mM phenylmethylsulfonyl fluoride (PMSF), 10 μg/mL pepstatin A, 1 mM benzamide, and 10 μg/mL leupeptin. For RNase A treatment, 400 μg/mL RNase A (Nacalai, Kyoto, Japan) was added to the lysis buffer, and the mixture was incubated for 30 min at 37°C. Then, a sample buffer comprising 0.35 M Bis-tris (pH 6.5), 2% SDS, and 10% glycerol were added. An RNA purification kit (Ambion, Austin, TX, USA) was added to 1/4 vol. of the cell lysates, and 100 mM dithiothreitol (DTT) was subsequently added. The processed samples were analyzed using 10% NuPAGE^TM^ gel (Invitrogen, Waltham, MA, USA) with a buffer comprising 50 mM 3-(*N*-morpholino) propanesulfonic acid (MOPS) (pH 7.7), 50 mM Tris base, 0.1% SDS, and 1 mM ethylenediaminetetraacetic acid (EDTA). The dried gels were placed in a lightproof case and an imaging plate (Fujifilm, Tokyo, Japan) was exposed to them for 3 days. The imaging plate was analyzed using a Fujifilm BAS-2500 system.

### Cell culture

HEK293T cells (RIKEN BRC, Ibaraki, Japan) (approximately 5.0 × 10^5^ cells) were seeded in 6-well dishes coated with collagen (Cellmatrix Type I-C; Nitta Gelatin, Osaka, Japan) and cultured in Dulbecco’s modified Eagle’s medium (DMEM; Nacalai) supple­mented with 10% fetal bovine serum (NICHIREI, Tokyo, Japan; Cat. No. 175012, Lot. No. 20C00F) at 37°C under 5% CO_2_. After 24 h, transfection was performed using polyethylenimine (PEI) Max (Polysciences, Warrington, PA, USA) with 1.25 μg of plasmid DNA. After a further 24 h, the cells were collected for analysis. For the harringtonin pulse-chase experiments, 1 μg/mL harringtonin was added to the HEK293T cells after 22 h of translation, and their lysates were recovered at the indicated times.

### Immunoblotting

**Fig. 1:**
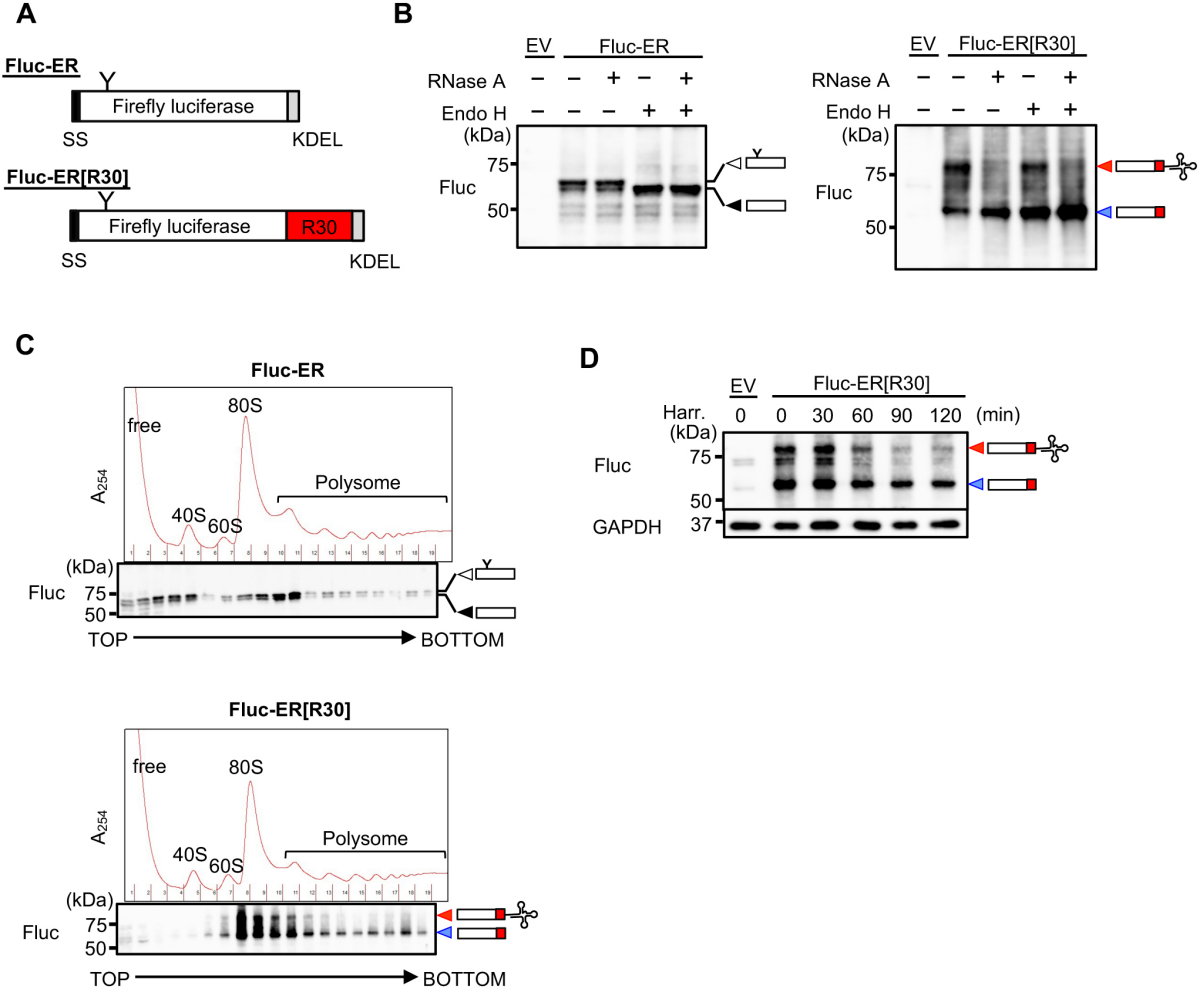
Fluc-ER[R30] causes translational arrest (A) Schematic diagrams of the ER-localized firefly luciferase (Fluc-ER) and Fluc-ER fused with 30 Arg residues at the C-terminus (Fluc-ER[R30]). Their N- and C-termini harbor a calreticulin-derived SS and KDEL, respectively. “Y” indicates an N-glycosylation site (N197 in Fluc sequence). (B) Lysates derived from HEK293T cells transiently expressing Fluc-ER or Fluc-ER[R30] were treated with 40 μg/mL RNase A and/or 2 units/μL endoglycosidase H (Endo H) to investigate their translational arrest and N-glycosylation levels. They were then subjected to immunoblot analysis using NuPAGE^TM^. The black, white, red, and blue arrowheads indicate unmodified full-length, glycosylated full-length, translationally arrested intermediates harboring covalently attached tRNA, and tRNA-removed arrested products. EV: empty vector. (C) Lysates derived from HEK293T cells transiently expressing Fluc-ER or Fluc-ER[R30] were analyzed by sucrose gradient centrifugation (10%–50%). The polysomal status was detected by measuring the absorbance at 254 nm. Each fraction was analyzed using an anti-Fluc antibody. The arrowheads are described in the legends for (B). (D) Time-course analysis of the translational arrest of Fluc-ER[R30]. HEK293T cells transiently expressing Fluc-ER[R30] were treated with 1 μg/mL harringtonin (Harr.) to prevent further initiation of translation. The lysates were then analyzed with the indicated antibodies using NuPAGE^TM^. The arrowheads are described in the legend for (B).

For NuPAGE^TM^ analysis, the cells were lysed on ice for 30 min in lysis buffer A, comprised of 0.35 M Bis-tris (pH 6.5), 2% SDS, 10% glycerol, 20 μM MG-132, 1 mM PMSF, 10 μg/mL pepstatin A, 1 mM benzamide, and 10 μg/mL leupeptin. Four aliquots of the sample buffer, comprised of 0.35 M Bis-tris (pH 6.5), 2% SDS, 10% glycerol, and RNA secure (Ambion), were added to 1/4 vol. of the cell lysates. This was followed by the addition of 100 mM DTT. The proteins were separated on an 8% or 10% NuPAGE^TM^ Bis-tris gel comprising 0.35 M Bis-tris (pH 6.5) with MOPS buffer. After electrophoresis, the proteins were transferred onto a polyvinylidene difluoride (PVDF) membrane (Merck, Darmstadt, Germany). Each protein was detected using the indicated antibodies. The PVDF membranes were developed using Amersham ECL western blotting detection reagent (GE Healthcare, Chicago, IL, USA) or Amersham ECL prime western blotting detection reagent (GE Healthcare) using a ChemiDoc^TM^ imager (Bio-Rad, Hercules, CA, USA) between 1 s and 60 min. In some cases, the chemiluminescence of the PVDF membranes was detected using an ImageQuant^TM^ LAS 4000 system (Fujifilm) between 1 s and 60 min (Fig. 1B, C, D). When the cell lysates were treated with RNase A or Endo H, 40 μg/mL RNase A (Nacalai) or 2 units/L Endo H (NEB, Ipswich, MA, USA) was added, and the mixture was incubated for 30 min at 37°C. For normal Laemmli SDS–polyacrylamide electrophoresis (SDS-PAGE), the cells were lysed for 30 min on ice in lysis buffer B, comprised of 50 mM Tris-HCl (pH 8.0), 150 mM NaCl, 1% Triton-X, 0.5% SDS, 20 μM MG-132, 1 mM PMSF, 10 μg/mL pepstatin A, 1 mM benzamide, and 10 μg/mL leupeptin. They were then analyzed using 8% or 10% Laemmli SDS-PAGE gels. After electrophoresis, the procedures were the same as those described above.

For phosphorylation assay, the cells were lysed for 30 min on ice in lysis buffer C, comprised of 50 mM Tris-HCl (pH 8.0), 150 mM NaCl, 1% Triton-X, 0.5% SDS, 20 μM MG-132, 1 mM PMSF, 10 μg/mL pepstatin A, 1 mM benzamide, 10 μg/mL leupeptin and Phos-STOP (Roche, Basel, Switzerland). They were then analyzed using 6% Laemmli SDS-PAGE gels for PERK or 4.5% Phos-tag gels, comprised of 0.375 M Tris-HCl (pH 8.8), 0.1% SDS, 0.1 mM MnCl_2_, and 12.5 μM Phos-tag (Wako, Osaka, Japan) for IRE1α.

### Polysome analysis

HEK 293T cells (approximately 3.0 × 10^6^ cells) were seeded on a 10 cm^2^ dish coated with collagen. After 24 h, they were transfected with plasmid DNA (7.5 μg). After a further 24 h, the cells were washed once with 10 mL of phosphate-buffered saline (PBS). The cells were lysed on ice for 30 min in 500 μL of lysis buffer C, comprised of 20 mM 4-(2-hydroxyethyl)-1-piperazineethanesulfonic acid/potassium hydroxide (HEPES-KOH) (pH 7.5), 100 mM KCl, 10 mM MgCl_2_, 0.25% NP-40, 20 μM MG-132, 1 mM PMSF, and a protease inhibitor cocktail (Nacalai). The cell lysate was centrifuged at 10,000 × *g* for 10 min at 4°C. The supernatant (500 μL) was then layered onto a 14 mL 10–50% sucrose gradient comprising 20 mM HEPES-KOH, 100 mM KCl, and 10 mM MgCl_2_. The samples were centrifuged at 35,000 rpm (200,000 × *g*) for 190 min at 4°C in a SW-40Ti rotor (Beckman Coulter, Brea, CA, USA). After centrifugation, the ribosomal distribution was estimated by measuring the optical absorbance at 254 nm. The proteins in each fraction were precipitated with trichloroacetic acid (TCA), as described previously (Shao and Hegde, 2014). The proteins were then analyzed by immunoblotting with NuPAGE^TM^ using MOPS buffer.

### Digitonin fractionation

The HEK293T cells were transfected with pcDNA3.1(+)_Fluc-ER (R30) and harvested using trypsin–EDTA. The cells were then suspended in DMEM and precipitated by centrifugation at 1,000 × *g* for 5 min at 4°C. After washing with ice-cold PBS, the cells were lysed on ice for 5 min in lysis buffer D, comprised of 50 mM HEPES-KOH (pH 7.5), 25 μg/mL digitonin (Wako), 150 mM KOAc, 2.5 mM Mg(OAc)_2_, 20 μM MG-132, 1 mM PMSF, 10 μg/mL pepstatin A, 1 mM benzamide, and 10 μg/mL leupeptin. The lysates were then centrifuged at 3,000 × *g* for 3 min at 4°C. The supernatants were obtained as cytosol fractions, and the pellets were washed and suspended again on ice for 5 min in lysis buffer E, comprised of 50 mM HEPES-KOH (pH 7.5), 1% TritonX-100, 0.5% deoxycholate, 500 mM KOAc, 2.5 mM Mg(OAc)_2_, 20 μM MG-132, 1 mM PMSF, 10 μg/mL pepstatin A, 1 mM benzamide, and 10 μg/mL leupeptin. The lysates were then centrifuged at 8,000 × *g* for 3 min at 4°C, and the supernatants were obtained as membrane fractions. The proteins in the resultant fractions were precipitated with TCA, dissolved in a smaller volume of lysis buffer E, and analyzed by immunoblotting with NuPAGE^TM^ using MOPS buffer.

### Immunoprecipitation

The HEK293T cells were transfected with pcDNA_Fluc-ER or pcDNA_Fluc-ER[R30]. After 24 h, the cells were lysed on ice for 5 min in IP buffer A, comprised of 50 mM HEPES-KOH (pH 7.5), 100 mM KCl, 10 mM MgCl_2_, 20 mg/mL dodecylmaltoside, 20 μM MG-132, 1 mM PMSF, 10 μg/mL pepstatin A, 1 mM benzamide, and 10 μg/mL leupeptin. The cell lysates were centrifuged at 17,700 × *g* for 20 min at 4°C, and the supernatant was precleared with equilibrated protein G-Sepharose 4 Fast Flow (GE Healthcare) for 30 min at 4°C. The supernatant was then incubated with 5 μg anti-Fluc antibody for 1 h at 4°C. Then, the protein G-Sepharose was added and the mixture was incubated for a further 30 min. The samples were then centrifuged at 2,000 × *g* for 30 s at 4°C, and the supernatant was removed. The beads were washed four times with IP buffer and then incubated with 1 × sample buffer for 30 min at 37°C.

### Statistical analysis

Quantification was performed using Multi Gauge ver.3.1 (Fujifilm) or Image Lab ver.6.0 software. One-way ANOVA with Tukey’s multiple comparison test was performed on data generated from n ≥ 3 biological replicates using GraphPad Prism version 8.00 software (GraphPad Software, San Diego, CA, USA). Results were deemed significant if *P* < 0.05, and were denoted *P < 0.05, **P < 0.01, and ***P < 0.001.

## Results

### Design of an ER-destined translational arrest protein (ER-TAP)

To clog translocons in mammalian cells, we designed an ER-TAP by attaching translational arrest motifs to model SPs at the C-terminus. As a model SP, we used Fluc-ER in which firefly luciferase was fused in-frame with the SS derived from calreticulin at the N-terminus (Nakamura *et al.*, 2011). An arginine (Arg, R) repeat sequence was used for the translational arrest sequence because it reportedly causes translational slowdown (Lu and Deutsch, 2008). We examined the translational arrest capability of Arg-repeat motifs ranging from 0 to 30 Arg residues. In the experiment, the Arg-repeat motifs were inserted between enhanced GFP (EGFP) and mCherry in-frame (the insertion segments were adjusted to 30 amino acids by replacing Arg with alanine (Ala, A) (Fig. S1A, B). These were then translated with an *in vitro* translation (IVT) system using a rabbit reticulocyte lysate. As a result, 30 consecutive Arg residues (R30) were found to induce strong translational arrest, judging by the appearance of an RNase A treatment-sensitive intermediate composed of covalently attached tRNA and polypeptide synthesized until the arresting point (Fig. S1C, upper left panel). We then attached the R30 motif to the C-terminus of Fluc-ER and named it Fluc-ER[R30] (Fig. 1A). As expected, the transiently expressed Fluc-ER[R30] predominantly existed as translationally arrested intermediates in the HEK293T cells (Fig. 1B, right panel). Furthermore, polysome analysis of the protein revealed that almost all the Fluc-ER[R30] proteins were associated with ribosomes, whereas Fluc-ER was distributed in lighter fractions in addition to the ribosome fractions (Fig. 1C). Importantly, the level of the translationally arrested intermediates did not decrease even after 30 min of treatment with the translational initiation inhibitor, harringtonin *in vivo*, although the level of intermediates gradually decreased in the following chase period (Fig. 1D). Taken together, R30 motif induced a strong translational arrest *in vitro* and *in vivo*.

**Fig. 2:**
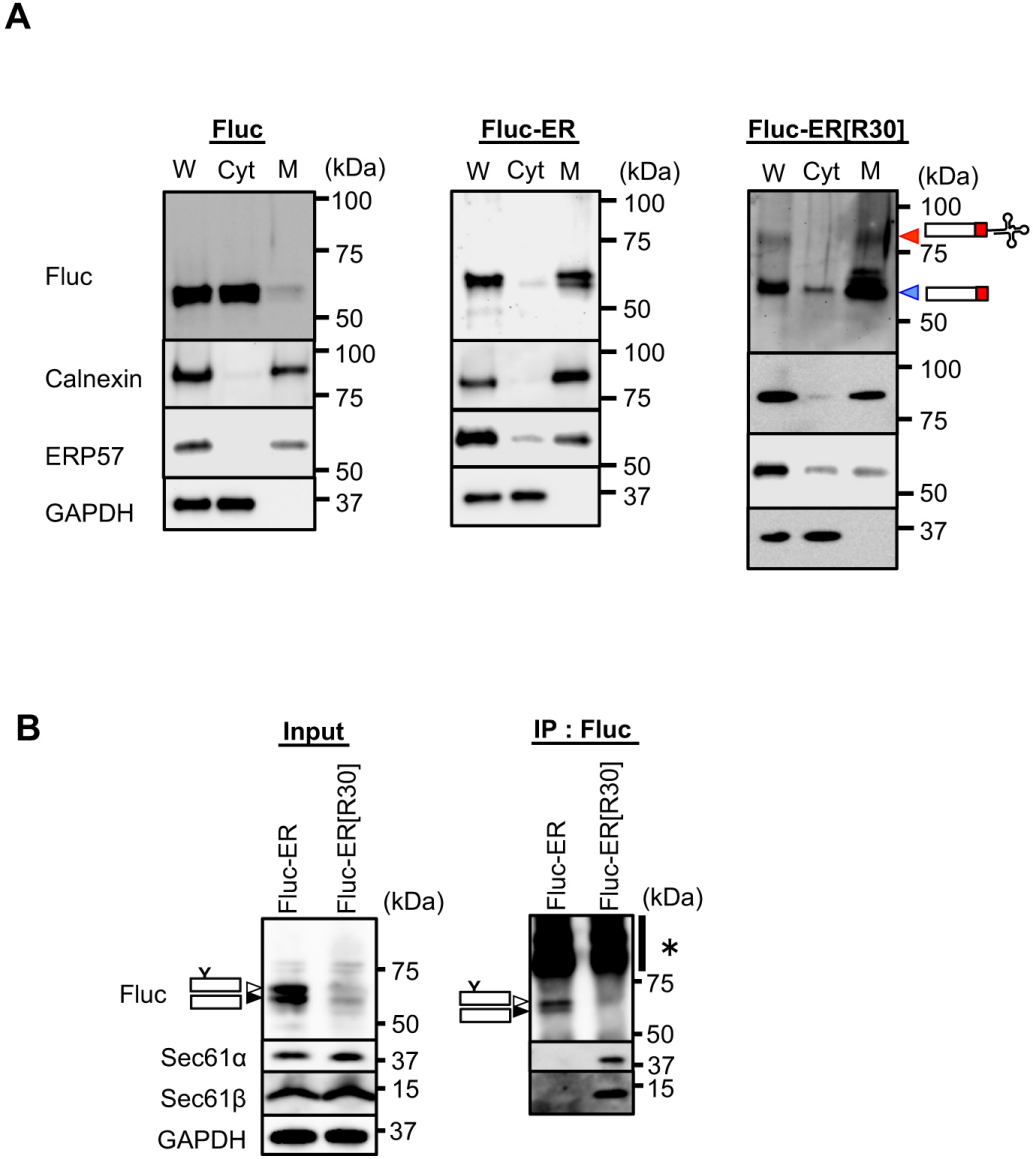
Fluc-ER[R30] co-translationally localized on the ER translocon (A) Lysates derived from HEK293T cells transiently expressing Fluc, Fluc-ER, Fluc[R30] or Fluc-ER[R30] were fractionated into cytosol (Cyt) and membrane (M) fractions using 25 μg/mL digitonin. Their fractions with the corresponding whole cell lysate (W) were then analyzed with NuPAGE^TM^ and the indicated antibodies. (B) Fluc-ER or Fluc-ER[R30] transiently expressed in HEK293T cells were immunoprecipitated with an anti-Fluc antibody. These immunoprecipitants were analyzed by immunoblotting with NuPAGE^TM^ and the indicated antibodies. The arrowheads are described in the legend for Fig. 1B. The asterisk indicates non-specific band.

We next examined the subcellular distribution of Fluc-ER[R30] (Fig. 1A and Fig. S2A). Biochemical cell fractionation using digitonin showed that the translationally arrested Fluc-ER[R30] was predominantly partitioned in the membrane fraction as Fluc-﻿ER, whereas cytosolic control, Fluc was predominantly partitioned in the cytosol fraction (Fig. 2A and Fig. S2B). Importantly, Fluc-ER[R30] was associated with translocon components Sec61α and Sec61β, whereas Fluc-ER was not (Fig. 2B). Collectively, we concluded that Fluc-ER[R30] exists as translationally arrested intermediates at translocons in the ER membrane (Fluc-ER[R30] is described as ER-TAP in the later part).

### SS selection is exaggerated in the presence of ER-TAP

**Fig. 3:**
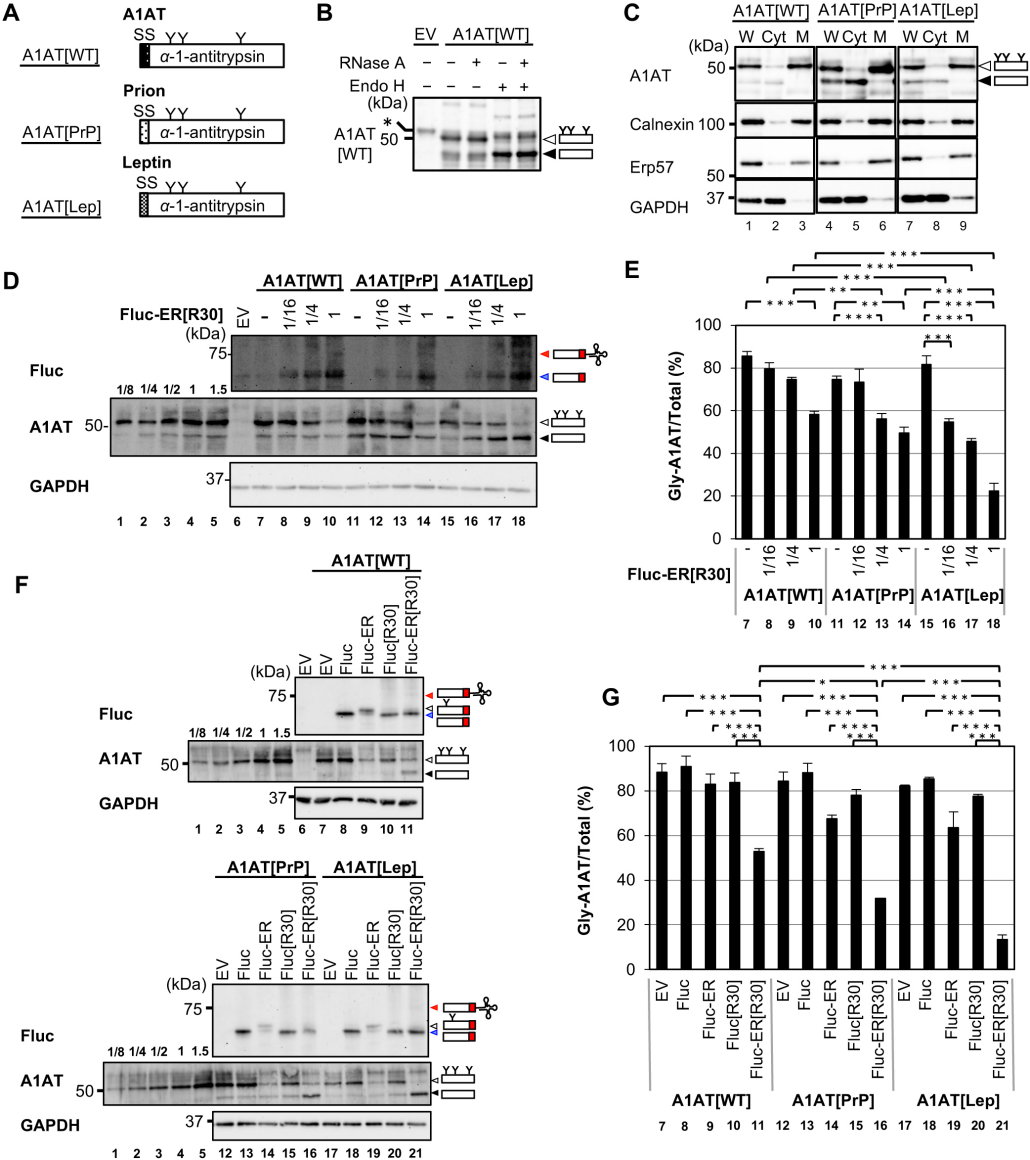
Secretory proteins harboring weak SS are predisposed to be repelled from the ER in the presence of ER-TAP (A) Schematic diagrams of α-1-antitrypsin (A1AT[WT]) variants. A1AT[PrP] and A1AT[Lep] harbored weak SS from prions and leptin, respectively, instead of the intact SS of A1AT[WT]. “Y” indicates N-linked glycosylation sites in the A1AT sequence (N70, N107, and N271). (B) Lysates derived from HEK293T cells transiently expressing A1AT[WT] were treated with 40 μg/mL RNase A and/or 2 units/μL Endo H to investigate their translational arrest and N-glycosylation levels, respectively. They were then subjected to immunoblot analysis using NuPAGE^TM^. The black, and white arrowheads indicate unmodified full-length and glycosylated full-length products, respectively. The asterisk indicates non-specific bands. (C) Lysates derived from HEK293T cells transiently expressing A1AT[WT], A1AT[PrP] and A1AT[Lep] were fractionated into cytosol (Cyt) and membrane (M) fractions using 25 μg/mL digitonin. Their fractions with the corresponding whole cell lysate (W) were then analyzed using an SDS-PAGE gel and the indicated antibodies. The arrowheads are described in the legend for (B). (D) The A1AT variants and a serial diluted amounts of Fluc-ER[R30] (1/16, 1/4, and 1) were transiently coexpressed in HEK293T cells, and their lysates were analyzed by immunoblotting with normal Laemmli SDS-PAGE gel (for A1AT, GAPDH), or with NuPAGE^TM^ (for Fluc). Different volumes of A1AT samples were loaded for calibration (lanes 1–5; 1/8, 1/4, 1/2, 1, and 1.5 volumes). The black, white, red, and blue arrowheads indicate unmodified full-length, glycosylated full-length, translationally arrested intermediates harboring covalently attached tRNA, and full-length or tRNA-removed arrested products, respectively. (E) The ratios of glycosylated A1AT in (D) were analyzed using one-way ANOVA with Tukey’s multiple comparison test (*P < 0.05, **P < 0.01, ***P < 0.001; n = 4). The lane numbers correspond to those indicated in the legend for (D). The band intensities were quantified by standard curve based on lanes 1–5 in (D). (F) A1AT variants were coexpressed with Fluc variants in HEK293T cells. The lysates were analyzed by immunoblotting, and the arrowheads are described in the legend for (D). (G) The ratios of glycosylated A1AT in (F) were analyzed using one-way ANOVA with Tukey’s multiple comparison test (ns, not significant; *P < 0.05, ***P < 0.001; n = 3). The lane numbers correspond to (F). The band intensities were quantified by standard curve based on lanes 1–5 in (F).

Given that ER-TAP obstructs translocons, we speculated that the expression of this protein may compromise the translocation efficiency of other SPs. To test this possibility, we evaluated the effect of ER-TAP/Fluc-ER[R30] on the translocation of a model SP—i.e., α1-antitrypsin (A1AT)—by measuring its glycosylated form. As shown in Fig. 3A and B, A1AT has three asparagine (N)-﻿linked glycosylation sites, which can be removed by endoglycosidase H (Endo H) treatment. Given that N-linked glycosylation occurs after the translocation of SPs into the ER, the translocation efficiency of A1AT can be determined by measuring the ratio between the unglycosylated and glycosylated forms. As shown in Fig. 3D and E, the translocation efficiency of A1AT was 86% (lane 7). This value was modestly decreased to 58% when an excessive amount of Fluc-ER[R30] was co-expressed with A1AT (Fig. 3D and E, lanes 8–10). A previous report suggests that the translocation efficiencies of SPs depend on their SS (Kim *et al.*, 2002). Furthermore, a group of proteins harboring weak SS, including prion protein and leptin, have been shown to not translocate into the ER under ER stress conditions due to compromised translocation capacity (Kang *et al.*, 2006). This inspired us to examine the effect of ER-TAP/Fluc-ER[R30] in the translocation of proteins harboring weak SS. For this purpose, we changed the SS of A1AT to weak SS derived from prion protein or leptin (A1AT[PrP], A1AT[Lep]) (Fig. 3A). As shown in Fig. 3C, the untranslocated species of A1AT[PrP] and A1AT[Lep] were more evident than A1AT[WT], as expected (lanes 1, 4, and 7). Biochemical cell fractionation using digitonin showed that almost all glycosylated and unglycosylated A1AT were partitioned in the membrane fraction and cytosol fraction, respectively (Fig. 3C, lanes 5, 6 and 8, 9). This again confirmed that the lower bands were untranslocated species. We then determined whether the expression of ER-TAP/Fluc-ER[R30] would affect the translocation efficiencies of A1AT[PrP] and A1AT[Lep]. As shown in Fig. 3D and E, the ER translocation efficiency of A1AT[PrP] and A1AT[Lep] were more compromised than that of A1AT[WT] when Fluc-ER[R30] was co-expressed (lanes 7–10, 11–14, and 15–18), which indicates that weak SS-harboring proteins are more severely repelled under the ER-TAP/Fluc-ER[R30]-expressed condition. This SS-dependent repul­sion was triggered by translocon obstruction because expres­sion of Fluc, ER-targeted Fluc (Fluc-ER) or cytosolic Fluc with C-terminal translational arrest motif (Fluc[R30]) did not affect or modestly compromised translocation efficiency of A1AT variants (Fig. 3F and G, lanes 7–11, 12–16, and 17–21). These results indicate that SS selection is exaggerated under limited translocation capacity.

### Translocon clogging is specifically sensed by an ER stress sensor inositol requiring enzyme 1α (IRE1α)

**Fig. 4:**
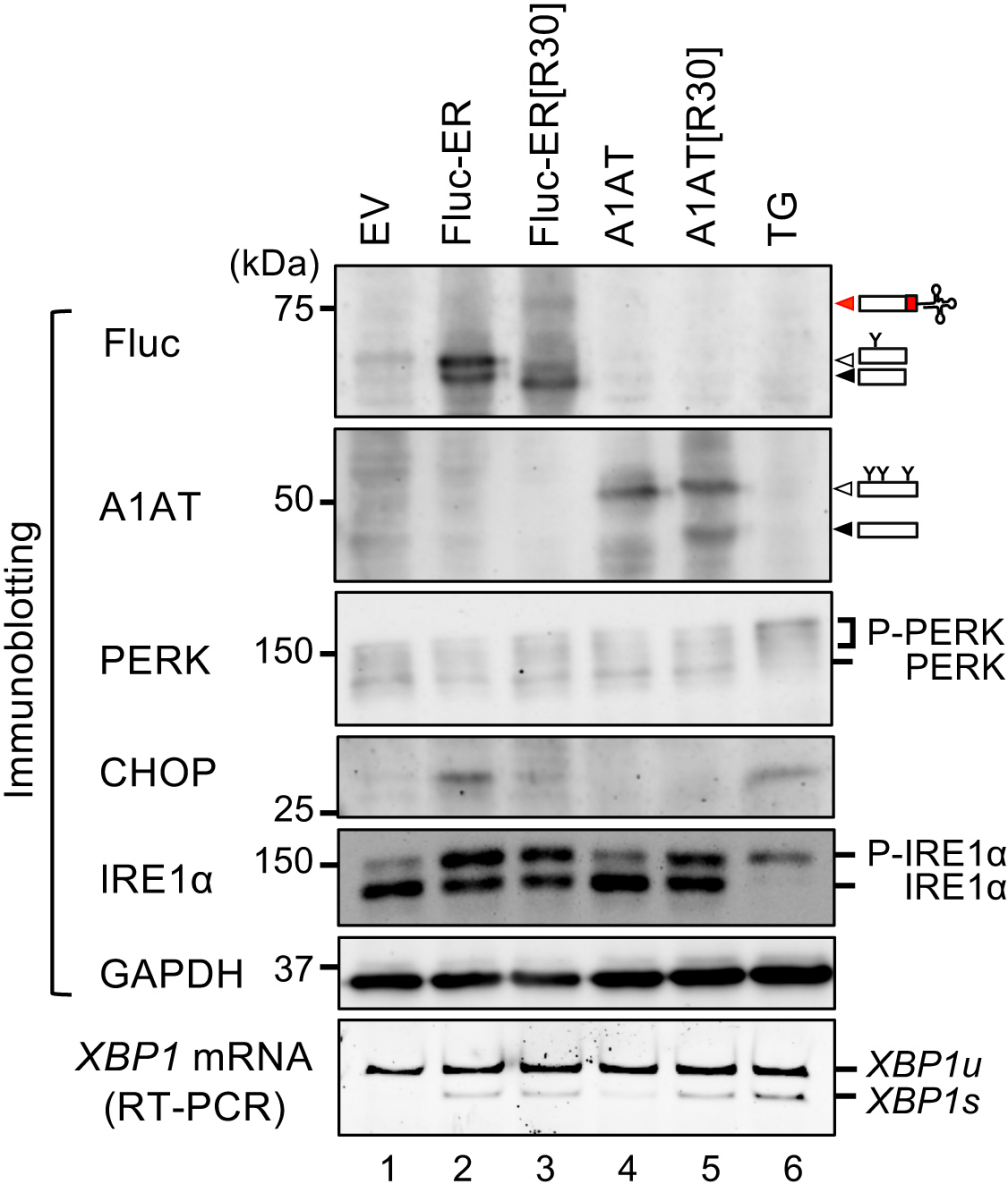
Translocon clogging is specifically sensed by IRE1α but not PERK Fluc-ER variants and A1AT variants were transiently expressed in HEK293T cells. The lysates were then analyzed by immunoblotting or reverse transcription-PCR (RT-PCR). For the immunoblotting, Fluc and A1AT were analyzed using NuPAGE^TM^; PERK, CHOP, and GAPDH were analyzed using normal Laemmli SDS-PAGE gels; phosphorylated IRE1α level was analyzed using a Phos-tag SDS-PAGE gel. As positive control for activation of the IRE1α and PERK pathways, HEK293T cells were treated with thapsigargin (TG; 0.5 μg/ml) for 2 h. Then, the lysate was analyzed as described above. The black, white, and red arrowheads indicate unmodified full-length or tRNA-removed arrested products, glycosylated full-length products, and translationally arrested intermediates harboring covalently attached tRNA, respectively. EV: empty vector.

Perturbation of proteostasis in the ER is believed to be sensed by ER stress sensors. Therefore, we tested how these sensors respond to the translocon obstruction using the Fluc variants. In this experiment, activation status of ER stress sensors, PKR-like ER kinase (PERK) and IRE1α, were monitored by detecting the phosphorylation levels of these proteins and downstream events which are induced by the activation of the ER stress sensors. As the downstream events, splicing of *XBP1* mRNA and CHOP protein up-regulation were monitored for activation of IRE1α and PERK pathway, respectively. The phosphorylation of PERK and IRE1α were detected by super-shift of these proteins by Laemmli SDS-PAGE and Phos-tag PAGE, respectively (Yang *et al.*, 2010; Kanda *et al.*, 2016). As expected, both PERK and IRE1α pathways were activated upon over-expression of ER-destined protein, Fluc-ER (Fig. 4, lane 2; Fig. S3A–D), not upon over-expression of cytosolic Fluc or Fluc[R30] (Fig. S3A and B). This was presumably due to excess protein influx into the ER and long-term retention in the ER of the proteins mediated by ER-retention signal in Fluc-ER, which would overwhelm the protein-folding capacity in the ER. In contrast, over-expression of ER-TAP/Fluc-ER[R30] strongly activated the IRE1α pathway whereas PERK pathway was unresponsive (Fig. 4, lane 3; Fig. S3). In addition to Fluc-ER[R30], we also tested another translocon clogger A1AT[R30] and its control A1AT (Fig. S2B and C; Fig. 4; Fig. S3C and D). As shown in Fig. 4, expression of A1AT[R30] induced activation of the IRE1α pathway but not the PERK pathway, whereas expression of A1AT did not activate both pathways. The two independent translocon cloggers specifically activate the IRE1α pathway but not the PERK pathway. Taken together, we proposed that ER-translocon clogging specifically activates the ER stress sensor IRE1α but not PERK. This model is based on the observation that expression of translocon cloggers specifically activates the IRE1α pathway. Considering that the two independent translocon cloggers specifically activate the IRE1α pathway, it is likely that translocon clogging specifically activates the IRE1α pathway. However, this phenomenon does not rule out a secondary effect of the expression of translocon clogger. In the future, following confirmation that the interaction between IRE1α and translocon is inhibited by expression of the translocon cloggers, our model will become more reliable.

## Discussion

**Fig. 5:**
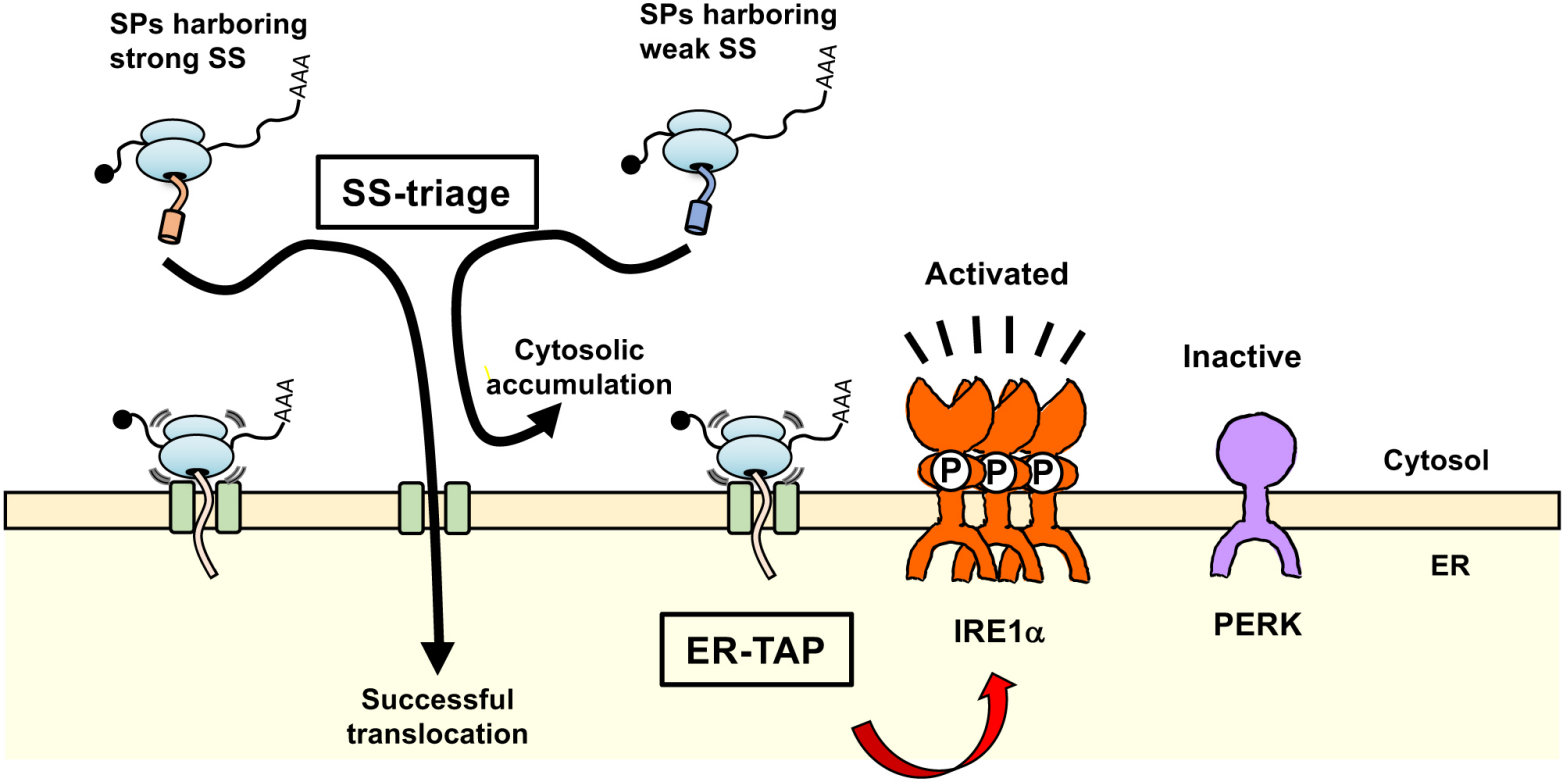
Decreased translocation capacity induced by ER-TAP triggers signal sequence-dependent triage and is sensed by IRE1α If protein translocation capacity in the ER is reduced by translocon obstruction, a series of SPs with weak SS are predisposed to be repelled from the ER, while SPs with strong SS pass through the ER translocon, which creates the SS-triage. The reduced translocation capacity is sensed by an ER stress sensor, IRE1α but not PERK. Given that the IRE1α pathway increased the translocation capacity by enlarging ER volume and up-regulation of the translocon components, ER expansions observed upon differentiation to secretory cells (i.e. plasma cells) are possibly mediated by a deficiency in the translocation capacity which is sensed by IRE1α.

We investigated the cellular response to ER-translocon clogging by developing an ER-TAP, Fluc-ER[R30]. We found that when the available ER-translocon levels were reduced by the expression of ER-TAP/Fluc-ER[R30], SS selection becomes more pronounced and SPs with weak SS cannot enter the ER (Fig. 5). This may be a cellular response that preferentially translocates some proteins to the ER during ER-translocon deficiency. In such a situation, it is advantageous for the cells to elicit a response that increases ER-translocation capacity. Identification of a group of proteins that are preferentially translocated to the ER in such situations would shed light on the significance of the cellular response that occurs upon translocon shortage (Fig. 5). Previously, Hegde’s group reported that a similar SS selection is triggered in situations where misfolded proteins accumulate in the ER (Kang *et al.*, 2006). In this case, SS selection is thought to be a cellular response to reduce the load on the protein folding machineries in the ER. The relationship between the two types of SS selection is currently unclear, but some causal relationship may be revealed in the future.

In addition to SS selection, we found that ER-translocon clogging is sensed by IRE1α but not PERK. Recently, it has been reported that activated IRE1α is inactivated by the binding of BiP at the ER-translocon (Li *et al.*, 2020). Based on the structure of the ribosome–translocon (Voorhees and Hegde, 2016; McGilvray *et al.*, 2020), IRE1α access of an ER-translocon engaging protein translocation is difficult, because IRE1α harbors a large domain in the cytosolic side (Ali *et al.*, 2011; Concha *et al.*, 2015; Harrington *et al.*, 2015). If so, IRE1α would be inactivated by free ER-translocons that are not engaged in protein translocation. When ER-TAP/Fluc-ER[R30] was expressed, it is likely that free ER-translocon levels were greatly reduced, which would facilitate the maintenance of the activated state of IRE1α. Previously, two different roles of translocon interaction with IRE1α were reported from the same group. One proposed that interaction of translocon with IRE1α is required for efficient activation of IRE1α (Plumb *et al.*, 2015). In contrast, another study proposed that interaction of IRE1α with translocon limits the activation of IRE1α (Sundaram *et al.*, 2017). Our results are consistent with that reported by the latter. In the ER stress response, activation of the IRE1α pathway has been reported to increase translocation capacity via induction of translocon component expression (Adamson *et al.*, 2016). Consistent with this, the IRE1α pathway but not the PERK pathway is reported to be indispensable for ER expansion upon differentiation of B cells to plasma cells (Reimold *et al.*, 2001; Gass *et al.*, 2008). In addition, it has been reported that IRE1α is specifically activated when the expression levels of Sec61α and Sec61γ, which are components of translocon, are decreased via CRISPRi (Adamson *et al.*, 2016). Taken together, we propose that cells interpret a decrease in free translocon levels as a shortage of the protein-translocation capacity relative to the protein influx into the ER. Then, this activates the IRE1α pathway to increase the translocation capacity by up-regulation of the components of the protein translocation machinery.

## Funding

This work was financially supported in part by the Japan Society of the Promotion of Science, KAKENHI (grant numbers JP24228002, JP26116006, JP17H01468, and JP15K21743 to K.Ko.); the Takeda Science Foundation (to H.Y. and K.Ko.); and the Ohsumi Frontier Science Foundation (to K.Ko.).

## Conflicts of Interest

The authors declare no competing or financial interests.

## Author Contributions

Conceptualization: A.S., K.Y., and K.Ko.; Methodology: A.S., R.K., M.O., K.Ka., A.T., and Y.K.; Investigation: A.S., R.K., K.Y., and K.Ko.; Data curation: A.S., R.K., K.Y., and K.Ko.; Visualization: A.S., R.K., K.Y., and K.Ko.; Writing: A.S., K.Y., and K.Ko.; Funding acquisition: H.K. and K.Ko.; Project administration: K.Ko.

## Abbreviations

A1AT: alfa 1-antitrypsin
DMSO: dimethyl sulfoxide
DTT: dithiothreitol
EGFP: enhanced green fluorescent protein
Endo H: endoglycosidase H
ER: endoplasmic reticulum
ER-﻿TAP: ER-destined translational arrest proteins
Fluc: firefly luciferase
Fluc-ER: ER-localized firefly luciferase
IRE1α: inositol requiring enzyme 1α
IVT: in vitro translation
Lep: leptin
ORF: open reading flame
PERK: protein kinase R (PKR)-like endoplasmic reticulum kinase
PrP: prion protein
PVDF: polyvinylidene difluoride
SPs: secretory proteins
SS: signal sequence(s)
XuC53: XBP1u pausing sequence
WT: wild-type
