# Supplementary material for: Signal sequence-triage is activated by translocon obstruction sensed by an ER stress sensor IRE1α
Source: Cell Struct Funct. 2023 Sep 28;48(2):211–21. doi: 10.1247/csf.23072 (PMC11496779; doi:10.1247/csf.23072)
Supplement: Supplementary file 1 — Supplementary Figures [file csf_48_23072_1.pdf]

Fig. S1

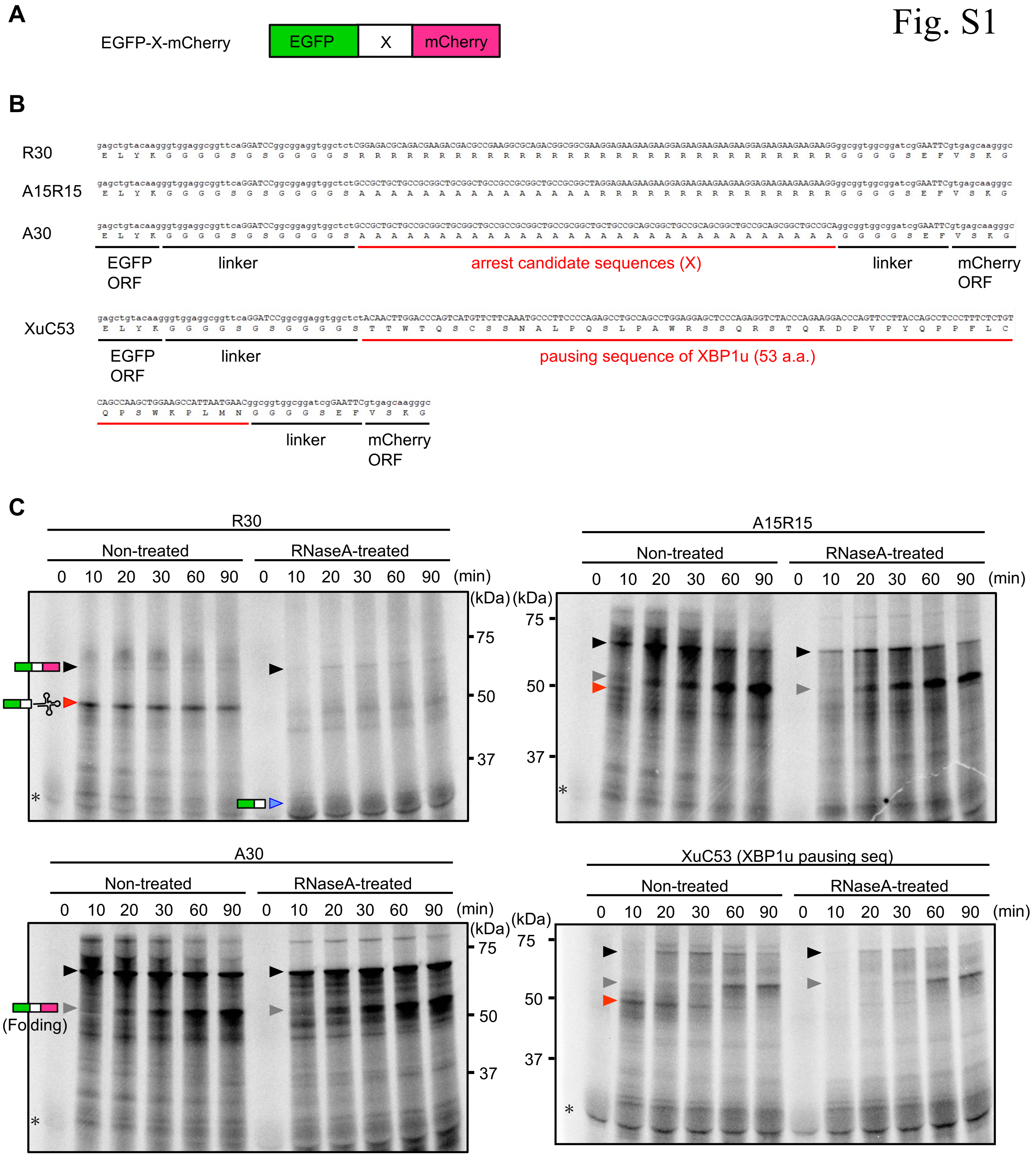
A
EGFP-X-mCherry
EGFP
X
mCherry
B
R30
A15R15
A30
EGFP ORF
linker
arrest candidate sequences (X)
linker
mCherry ORF
XuC53
EGFP ORF
linker
pausing sequence of XBP1u (53 a.a.)
linker
mCherry ORF
C
R30
Non-treated
RNaseA-treated
0 10 20 30 60 90 0 10 20 30 60 90 (min)
(kDa)
75
50
37
A15R15
Non-treated
RNaseA-treated
0 10 20 30 60 90 0 10 20 30 60 90 (min)
(kDa)
75
50
37
A30
Non-treated
RNaseA-treated
0 10 20 30 60 90 0 10 20 30 60 90 (min)
(kDa)
75
50
37
(Folding)
XuC53 (XBP1u pausing seq)
Non-treated
RNaseA-treated
0 10 20 30 60 90 0 10 20 30 60 90 (min)
(kDa)
75
50
37

Fig. S2

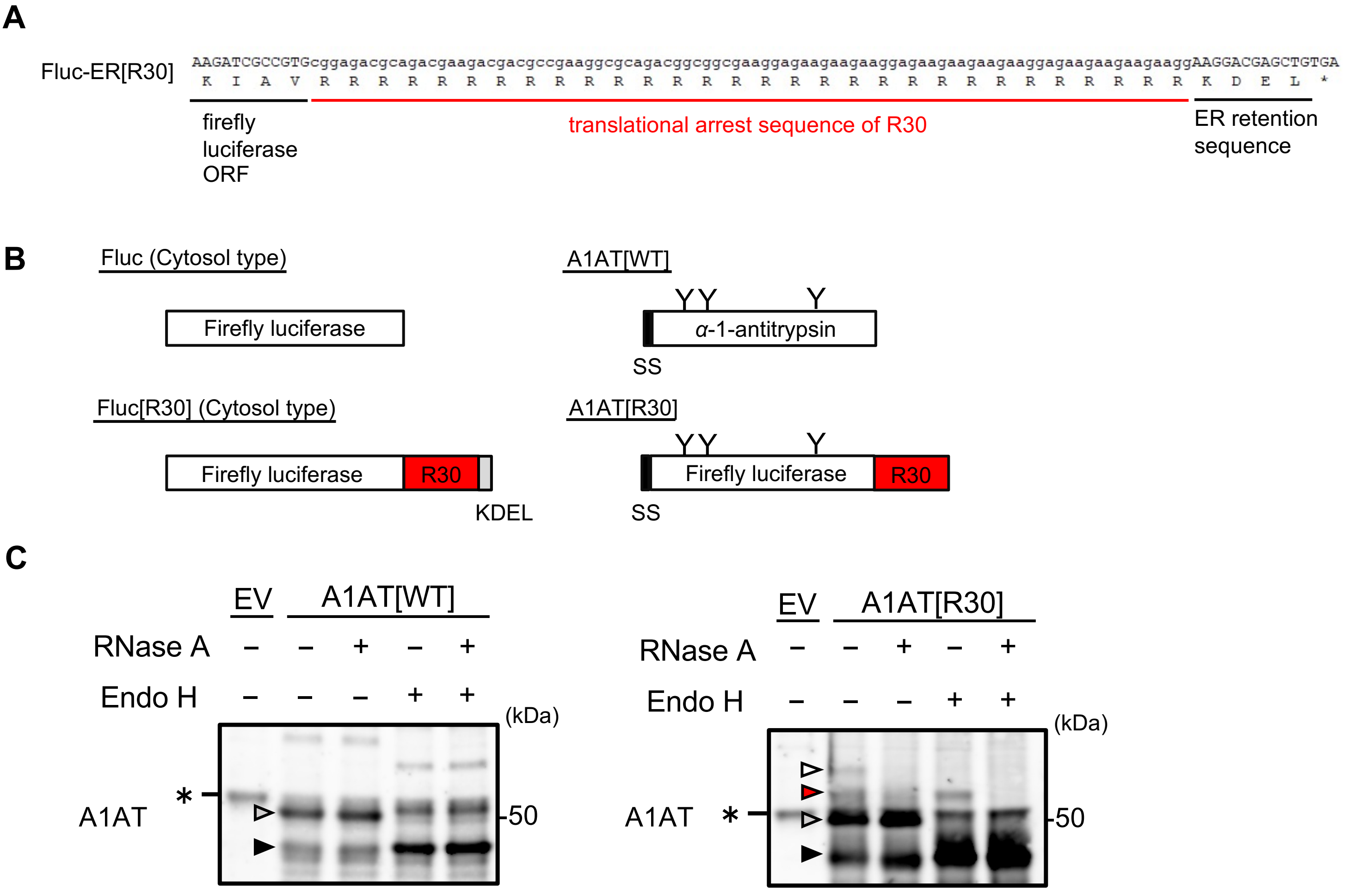

A
Fluc-ER[R30]
AAGATCGCCGTGcggagacgcagacgaagacgacgccgaaggcgcagacggcggcgaaggagaagaagaaggagaagaagaagaaggagaagaagaagaaggAAGGACGAGCTGTGA
K I A V R R R R R R R R R R R R R R R R R R R R R R R R R R R R R R K D E L *
firefly luciferase ORF
translational arrest sequence of R30
ER retention sequence
B
Fluc (Cytosol type)
Firefly luciferase
Fluc[R30] (Cytosol type)
Firefly luciferase
R30
KDEL
A1AT[WT]
α-1-antitrypsin
SS
A1AT[R30]
Firefly luciferase
R30
SS
C
EV
A1AT[WT]
RNase A – – + – +
Endo H – – – + +
(kDa)
A1AT
*
50
EV
A1AT[R30]
RNase A – – + – +
Endo H – – – + +
(kDa)
A1AT
*
50

Fig. S3

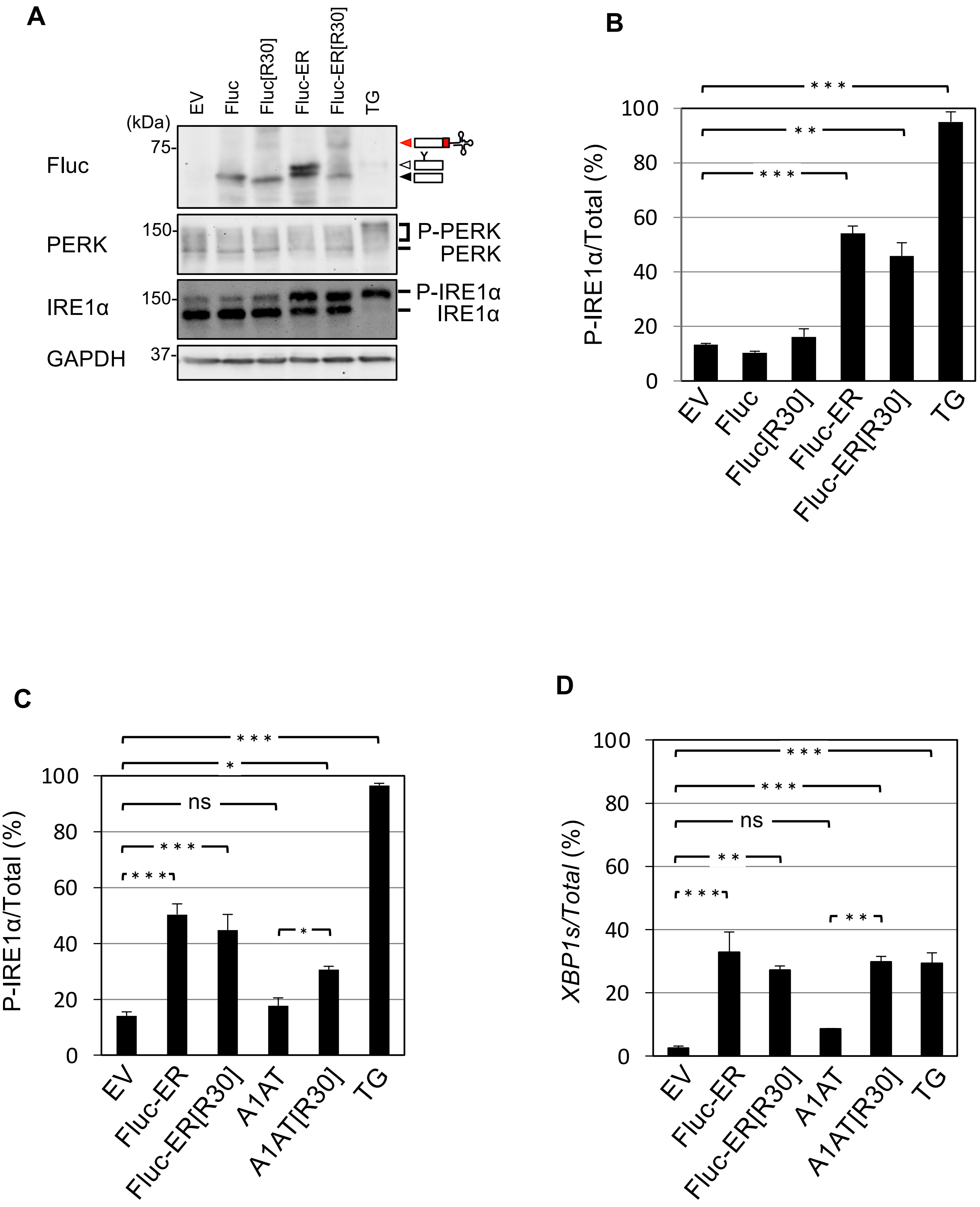

A
EV
Fluc
Fluc[R30]
Fluc-ER
Fluc-ER[R30]
TG
(kDa)
75-
Fluc
150-
PERK
P-PERK
PERK
150-
IRE1α
P-IRE1α
IRE1α
37-
GAPDH
B
P-IRE1α/Total (%)
100
80
60
40
20
0
EV
Fluc
Fluc[R30]
Fluc-ER
Fluc-ER[R30]
TG
* * *
* *
* * *
C
P-IRE1α/Total (%)
100
80
60
40
20
0
EV
Fluc-ER
Fluc-ER[R30]
A1AT
A1AT[R30]
TG
* * *
*
ns
* * *
* * * *
*
D
XBP1s/Total (%)
100
80
60
40
20
0
EV
Fluc-ER
Fluc-ER[R30]
A1AT
A1AT[R30]
TG
* * *
* * *
ns
* *
* * * *
* *

Supplementary figure legends

Figure S1. Thirty-Arg-residue motif triggers strong translational arrest.

(A) Schematic diagrams of the reporters for translational arrest. In the constructs, the arrest candidate sequences (indicated as “X”) were located between the enhanced green fluorescent protein (EGFP) and mCherry. **(B)** Nucleic acid and amino acid sequences around the X region. We tested 30 consecutive Arg residues (R30) and 15 consecutive Arg residues followed by 15 consecutive Ala residues (A15R15) as arrest candidates. The thirty consecutive Ala residues (A30) and pausing sequence in XBP1u (XuC53) constituted the negative and positive controls for translational arrest, respectively. **(C)** The arrest activities were tested by IVT using rabbit reticulocyte lysate. The indicated proteins were synthesized by the addition of the corresponding mRNAs to the IVT system. Translation was allowed to proceed for the indicated times. The resultant proteins were separated using NuPAGE™ and visualized by autoradiography. Note that the tRNA (~17 kDa) was covalently attached to the C-terminus of the arrested products. The arrested products shifted 17 kDa downward upon RNase treatment. The black, red, blue, and gray arrowheads indicate full-length products, translationally arrested intermediates harboring covalently attached tRNA, arrested products with tRNA removed, and folded full-length products (β-barrel proteins such as EGFP and mCherry retain their folded states even under SDS-containing buffer unless they are heat-denatured), respectively; the asterisks indicate non-specific bands. In the case of EGFP-XuC53-mCherry, the tRNA-removed arrested products were not detected because they were overlaid on nonspecific signals.

Figure S2. Detailed sequence of plasmid DNA used in the present study.

(A) Nucleic acid sequence and amino acid sequence around the translational arrest sequence in Fluc-ER[R30]. **(B)** Schematic diagrams of firefly luciferase (Fluc), Fluc fused with 30 Arg residues at the C-terminus (Fluc[R30]), A1AT, A1AT fused with 30 Arg residues at the C-terminus (A1AT[R30]). **(C)** Lysates derived from HEK293T cells transiently expressing A1AT or A1AT[R30] were treated with 40 μg/mL RNase A and/or 2 units/μL Endo H to investigate their translational arrest and N-glycosylation levels. They were then subjected to immunoblot analysis using NuPAGE™. The black, white, and red

arrowheads indicate unmodified full-length, glycosylated full-length, and translationally arrested intermediates harboring covalently attached tRNA. EV: empty vector. The asterisks indicate non-specific band.

Figure S3. Translocon clogging is specifically sensed by IRE1α but not PERK.

(A) Fluc-ER variants were transiently expressed in HEK293T cells and their lysates were then analyzed by immunoblotting. Fluc levels were analyzed using NuPAGE™; PERK and GAPDH levels were analyzed using normal Laemmli SDS-PAGE gels; IRE1α levels were analyzed using a Phos-tag SDS-PAGE gel. As positive control for activation of the IRE1α and PERK pathways, HEK293T cells were treated with TG (0.5 μg/ml) for 2 h. Then, the lysate was analyzed as described above. The black, white, and red arrowheads indicate unmodified full-length or tRNA-removed arrested products, glycosylated full-length products, and translationally arrested intermediates harboring covalently attached tRNA, respectively. **(B)** Ratios of phosphorylated IRE1α in Fig. S3(A) were analyzed using one-way ANOVA with Tukey's multiple comparison test (**$P < 0.01$, ***$P < 0.001$; n = 3). **(C)** Ratios of phosphorylated IRE1α in Fig. 4 were analyzed using one-way ANOVA with Tukey's multiple comparison test (ns, not significant; *$P < 0.05$, **$P < 0.01$, ***$P < 0.001$; n = 3). **(D)** Ratios of spliced form of *XBP1* mRNA in Fig. 4 were analyzed using one-way ANOVA with Tukey's multiple comparison test (ns, not significant; **$P < 0.01$, ***$P < 0.001$; n = 3).
